# NIH peer review percentile scores are poorly predictive of grant productivity

**DOI:** 10.7554/eLife.13323

**Published:** 2016-02-16

**Authors:** Ferric C Fang, Anthony Bowen, Arturo Casadevall

**Affiliations:** 1 Departments of Laboratory Medicine and Microbiology, University of Washington School of Medicine, Seattle, United States; 2 Department of Microbiology and Immunology, Albert Einstein College of Medicine, New York, United States; 3 Department of Molecular Microbiology and Immunology, Johns Hopkins Bloomberg School of Public Health, Baltimore, United States

**Keywords:** peer review, research funding, policy, national institute of health, grants, None

## Abstract

Peer review is widely used to assess grant applications so that the highest ranked applications can be funded. A number of studies have questioned the ability of peer review panels to predict the productivity of applications, but a recent analysis of grants funded by the National Institutes of Health (NIH) in the US found that the percentile scores awarded by peer review panels correlated with productivity as measured by citations of grant-supported publications. Here, based on a re-analysis of these data for the 102,740 funded grants with percentile scores of 20 or better, we report that these percentile scores are a poor discriminator of productivity. This underscores the limitations of peer review as a means of assessing grant applications in an era when typical success rates are often as low as about 10%.

**DOI:** http://dx.doi.org/10.7554/eLife.13323.001

## Introduction

Most funding agencies employ panels in which experts review proposals and assign scores to them based on a number of factors (such as expected impact and scientific quality). However, several studies have suggested significant problems with the current system of grant peer review. One problem is that the number of reviewers is typically inadequate to provide statistical precision (Kaplan et al., 2008). Researchers have also found considerable variation among scores and disagreement regarding review criteria (Mayo et al., 2006; Graves et al., 2011; Abdoul et al., 2012), and a Bayesian hierarchical statistical model of 18,959 applications to the NIH found evidence of reviewer bias that influenced as much as a quarter of funding decisions (Johnson, 2008).

Although there is general agreement that peer review can discriminate sound grant applications from those containing serious flaws, it is uncertain whether peer review can accurately predict those meritorious applications that are most likely to be productive. An analysis of over 400 competing renewal grant applications at one NIH institute (the National Institute of General Medical Sciences) found no correlation between percentile score and publication productivity of funded grants (Berg, 2013). A subsequent study of 1492 grants at another NIH institute (the National Heart, Lung and Blood Institute) similarly found no correlation between the percentile score and publication or citation productivity, even after correction for numerous variables (Danthi et al., 2014). These observations suggest that once grant applications have been determined to be meritorious, expert reviewers cannot accurately predict their productivity.

In contrast, a recent analysis of over 130,000 grant applications funded by the NIH between 1980 and 2008 concluded that better percentile scores consistently correlate with greater productivity (Li and Agha, 2015). Although the limitations of using retrospective publication/citation productivity to validate peer review are acknowledged (Lindner et al., 2015; Lauer and Nakamura, 2015), this large study has been interpreted as vindicating grant peer review (Mervis, 2015; Williams, 2015). However, the relevance of those findings for the current situation is questionable since the analysis included many funded grants with poor percentile scores (>40th percentile) that would not be considered competitive today. Moreover, this study did not examine the important question of whether percentile scores can accurately stratify meritorious applications to identify those most likely to be productive.

We therefore performed a re-analysis of the same dataset to specifically address this question. Our analysis focused on subset of grants in the earlier study (Li and Agha, 2015) that were awarded a percentile score of 20 or better: this subset contained 102,740 grants. This percentile range is most relevant because NIH paylines (that is, the lowest percentile score that is funded) seldom exceed the 20th percentile and have hovered around the 10th percentile for some institutes in recent years.

## Results

### Publication and citation productivity in relation to percentile score

**Figure 1.**
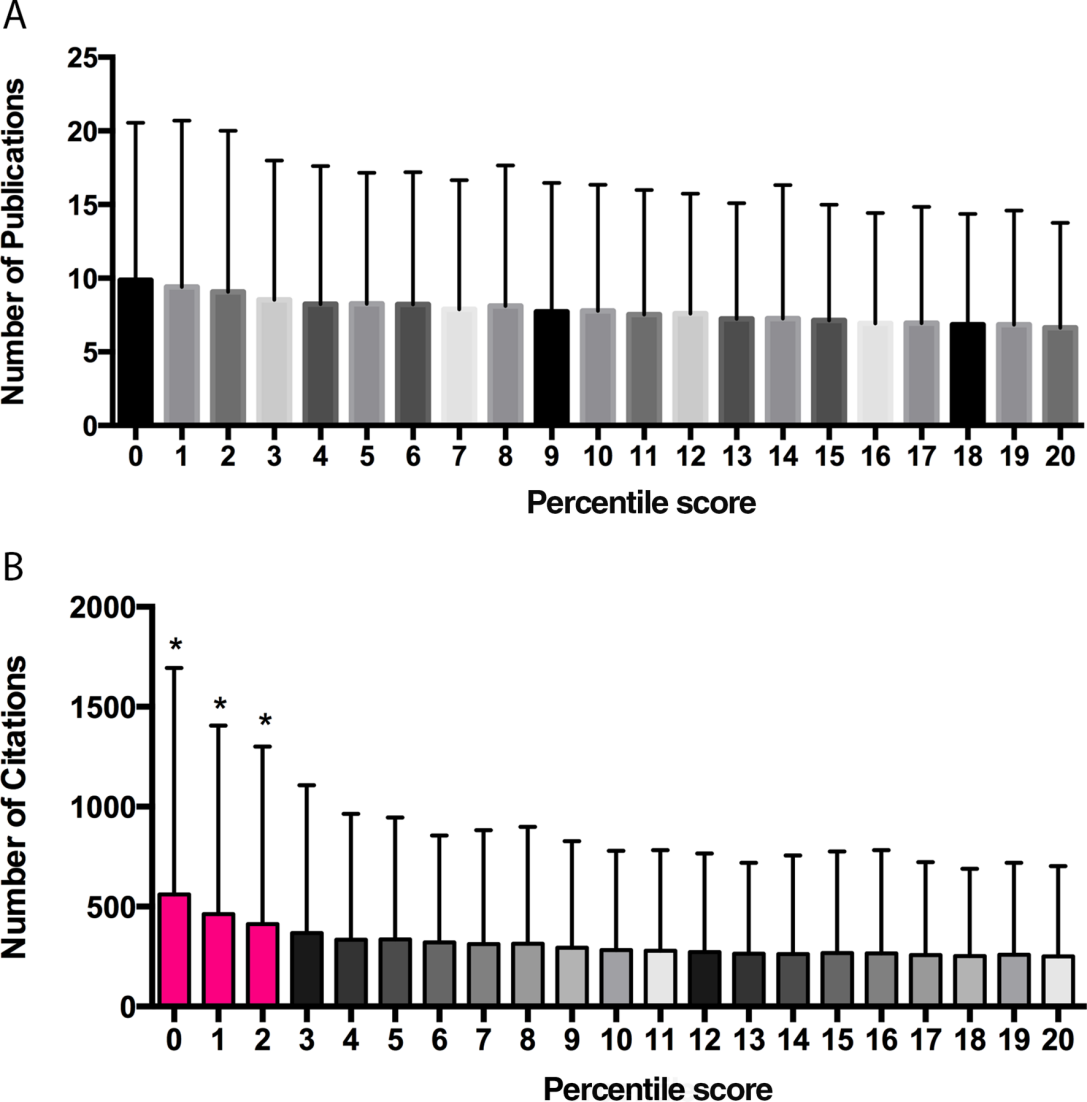
Publication and citation productivity in relation to percentile score. (**A**) The number of publications acknowledging support from grants within five years of grant approval (from PubMed) versus the percentile score: the bar shows the mean number of publications for all grants with that percentile score. (**B**) The number of citations that the papers in (**A**) received until the end of 2013 (data from Web of Science) versus the percentile score: the bar shows the mean number of citations for all grants with that percentile score. The lowest percentile scores are the most favorable. n = 102,740. Error bars = SDM. *Pink bars indicate significantly different from all cohorts of grants receiving poorer scores by one-way ANOVA. Black and gray bars do not differ significantly from their neighbors and are shown in different shades to allow easier visualization. **DOI:** http://dx.doi.org/10.7554/eLife.13323.002

A median of 6 publications was supported per grant. A plot of publication productivity versus percentile score (Figure 1A) shows a wide range of publication productivity at any given percentile score with no significant difference in productivity between any adjacent cohorts. A plot of citation productivity versus percentile score (Figure 1B) shows similar variability in productivity. Applications with percentile scores of 2 or better generated significantly more citations than applications receiving poorer scores; however, this was not true for applications receiving scores of 3 or higher. Although it was not possible to adjust citation numbers according to the year of award, a previous analysis of the dataset found that cohort effects relating to fiscal year did not influence the relationship between percentile score and productivity (Li and Agha, 2015). While our re-analysis confirms that there is a correlation between percentile score and publication or citation productivity for applications with scores in the top 20 percentiles, the correlation is quite modest (slope = −0.132 ± 0.005 publications and −9.6 ± 0.337 citations for each percentile score increment, r^2^ = 0.0078), suggesting that the overall ability of review groups to predict application success is weak at best. A random forest model indicated that only ~1% of the variance in productivity could be accounted for by percentile ranking (Figure 1—figure supplement 1), suggesting that all of the effort currently spent in peer review has a minimal impact in stratifying meritorious applications relative to what would be expected from a random ranking.

**Figure 1—figure supplement 1.**
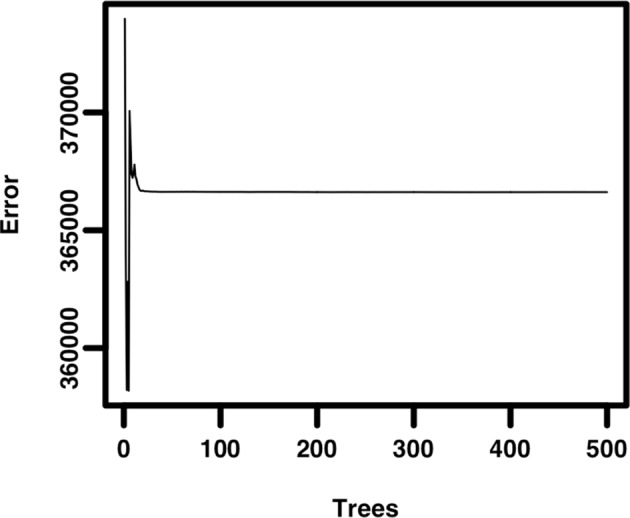
Random forest model of grant percentile score as a predictor of citation productivity. A non-parametric model was constructed with 500 trees to measure grant percentile score as a predictor of citation productivity. The results indicate that 0.98% of variance in productivity can be accounted for by percentile score. The mean of squared residuals converges to 366,620. **DOI:** http://dx.doi.org/10.7554/eLife.13323.003

### Percentile scores of grants stratified on the basis of citation and publication productivity

**Figure 2.**
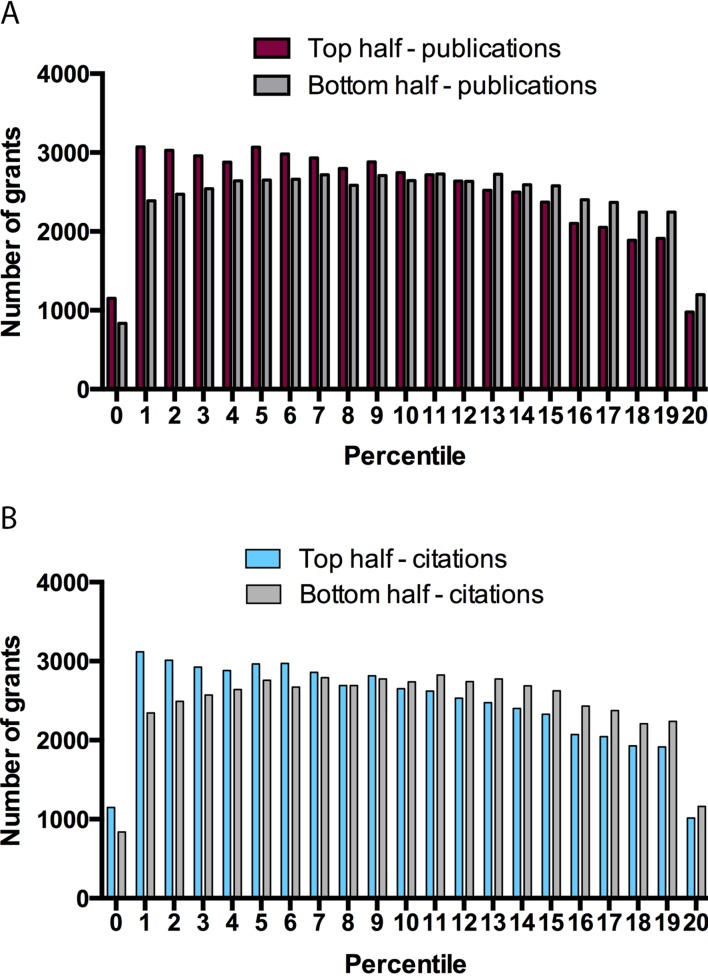
Grants stratified on the basis of publication and citation productivity for different percentile scores. Graphs showing, for percentile scores of 20 or better, the number of grants in the top half (left bar) and bottom half (right right) of grants on the basis of publications (**A**) and citations (**B**). Grants in the top half on the basis of publication productivity (**A**) had ≥ 6 publications: mean percentile score of top half 9.244 ± 5.583, median 9; mean percentile score of bottom half 9.947 ± 5.612, median 10. Grants in the top half on the basis of citation productivity (**B**) had ≥ 128 citations: mean percentile score of top half 9.242 ± 5.625, median 9; mean percentile score of bottom half 9.939 ± 5.571, median 10. Fewer grants received a percentile score of zero as a result of rounding to the nearest whole number, as well as a change in the NIH percentiling algorithm since 2009. **DOI:** http://dx.doi.org/10.7554/eLife.13323.004

This preceding analysis shows that percentile scores cannot accurately predict the subsequent productivity of a research grant in the critical 3–20 percentile range. This can be demonstrated by dividing the sample into equal halves based on publication productivity (the top 50.8% had ≥6 publications) and citation productivity (the top 49.99% had ≥128 citations). Substantial overlap is evident between percentile scores for grants in the upper and lower halves based upon both publication productivity (Figure 2A) and citation productivity (Figure 2B): for example, if only half of applications could be funded, a grant in the top half of applications based on citations would only have a 58% chance of being funded if decisions were based on percentile scores.

### Receiver operating characteristic (ROC) curve of grant peer review

**Figure 3.**
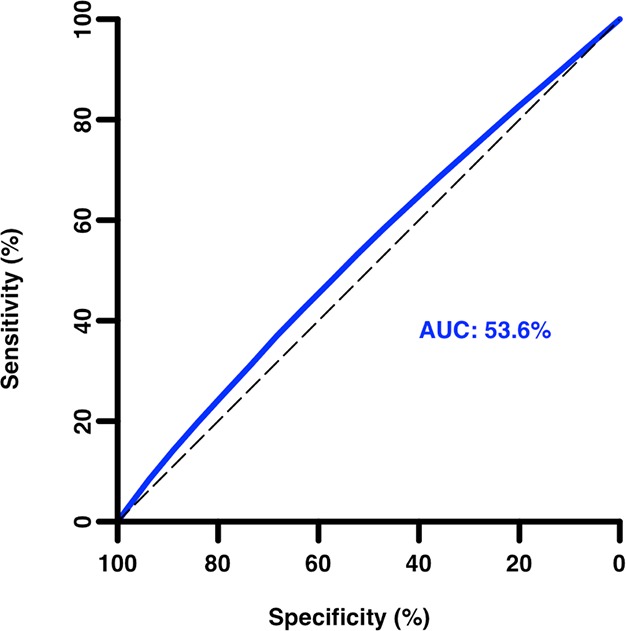
Receiver operating characteristic curve of grant percentile score as a predictor of citation productivity (low/high). Area under the curve (AUC) = 0.54 (95% confidence interval: 0.53–0.54) for citation productivity greater than the median. An AUC of 1.0 corresponds to a perfect test; an AUC of 0.5 indicates performance equivalent to random chance alone. **DOI:** http://dx.doi.org/10.7554/eLife.13323.005

An ROC curve (Figure 3) confirms the very poor discriminatory ability of percentile score for grants receiving scores in the 20th percentile or better, with an AUC (area under the curve) of only 0.54. Even an outstanding percentile score is no guarantor of project success, as 17% (334 of 1987) of grants with a percentile score of zero failed to produce any citations.

## Discussion

These observations suggest that despite the overall ability of reviewers to discriminate between extremely strong grant applications and the remainder, they have limited ability to accurately predict future productivity of meritorious applications in the range relevant to current paylines. This may contribute to a pervasive sense of arbitrariness with regard to funding decisions and dissatisfaction with the peer review system (Pagano, 2006; Fang and Casadevall, 2009; Costello, 2010; Germain, 2015). Perhaps most importantly, these findings contradict the notion that peer review can determine which applications are most likely to be productive. The excellent productivity exhibited by many projects with relatively poor scores and the poor productivity exhibited by some projects with outstanding scores demonstrate the inherent unpredictability of scientific research. The data also suggest that current paylines are inadequate to fund the most productive applications and that considerable potential productivity is being left on the table at current funding levels (Berg, 2011).

It should be noted that percentile scores and publication/citation productivity are not necessarily independent. Citation practices have evolved over time, and citations per paper approximately doubled between 1980 and 2004 (Wallace et al., 2009). This may have contributed to greater citation productivity for more recent grants, which would tend to have more favorable percentile scores than older ones, as paylines have declined over time, resulting in a correlation between percentile score and citation productivity that is unrelated to the discriminatory ability of the peer review process. In addition, applications that are funded despite scoring worse than the payline tend to be funded later in the fiscal year and are often subject to budgetary reductions that could adversely impact their future productivity. According to a report from the US Government Accountability Office (GAO), 13% of R01 applications funded in fiscal years 2003–2007 were exceptions with scores worse than the payline (GAO, 2009). This may be an additional contributing factor to the correlation between percentile score and publication and citation productivity that is not indicative of peer review discrimination.

These observations have important implications for the grant peer review system. If reviewers are unable to reliably predict which meritorious applications are most likely to be productive, then reviewers might save time and resources by simply identifying the top 20% and awarding funding within this group on a random basis or according to programmatic priorities. In this regard, we refer to our recent suggestion that the NIH consider a modified lottery system (Fang and Casadevall, 2014) and note that the New Zealand Health Research Council has already moved to a lottery system to select proposals for funding in its Explorer Grants program (Health Research Council of New Zealand, 2015).

In summary, while our analysis confirms that peer review has some ability to discriminate between the quality of proposals based on citation productivity over the entire range of percentile scores (Li and Agha, 2015), this does not extend to the critical range of percentile scores between 3 and 20, which is the most relevant subset of applications at current paylines. Furthermore, the best science is not necessarily receiving support under the present system since most applications in this percentile range, whether above or below current paylines, are indistinguishable with regard to citation productivity. The manner in which scarce research funds are allocated deserves greater attention.

## Methods

A database derived from 102,740 research project (R01) grants funded by the NIH from 1980 through 2008 was obtained from Danielle Li (Harvard University) and Richard Nakamura (NIH Center for Scientific Review). The data included the number of publications acknowledging grant support within 5 years of grant approval (from PubMed), the number of citations of those publications through 2013 (Web of Science) and the percentile score of the application (rounded to the nearest whole number). Parametric statistical analyses were performed in Prism (GraphPad Software, San Diego, CA). The receiver operating characteristic (ROC) and random forest analyses were performed using R (Vienna, Austria) and the pROC (Robin et al., 2011) and randomForest (Liaw and Wiener, 2002) packages.
